# Intact cerebrovascular autoregulation in patients with refractory status epilepticus due to sufficient anesthetic treatment on a neurointensive care unit: a prospective cohort study

**DOI:** 10.1186/s13054-018-2296-2

**Published:** 2019-01-09

**Authors:** Marco Meyer, Martin Juenemann, Tobias Braun, Ingo Schirotzek, Patrick Schramm

**Affiliations:** 1 Department of Geriatric, Jung-Stilling Hospital Siegen, Wichernstrasse 40, 57074 Siegen, Germany; 2 Department of Neurology, University Hospital Giessen and Marburg location Giessen, Klinikstrasse 33, 35385 Giessen, Germany; 3 Department of Neurology, University Hospital Giessen and Marburg Location Marburg, Baldingerstrasse, 35033 Marburg, Germany; 4 Department of Anesthesiology, Johannes Gutenberg-University, University Medical Hospital Mainz, Langenbeckstr. 1, 55131 Mainz, Germany

Status epilepticus (SE) is a serious emergency requiring immediate therapy to prevent severe seizure-related brain damage and secondary complications. Cerebrovascular autoregulation (CA) is a key component of cerebral hemostasis and is interictally compromised in epilepsy patients [1]. An impairment of CA may cause further neuronal damage due to fluctuations in cerebral perfusion pressure with consecutive cerebral edema or ischemia. We recently explored the time course of CA in patients with refractory SE and the need for deep analgo-sedation after failure of basic treatment with benzodiazepines and high-dose levetiracetam. All patients received analgo-sedation using propofol or midazolam and sufentanil with the aim of a burst-suppression pattern in electroencephalography. Mechanical ventilation was adapted to normocapnia and blood pressure to normal values. CA was calculated once daily for the first 4 days after the onset of SE by correlation of cerebral blood flow velocities (CBFVs) in both middle cerebral arteries measured with transcranial Doppler ultrasound and invasively measured arterial blood pressure. CA was expressed as the mean velocity index (Mx) as previously described [2]. Mx is a variable with no defined cut-off, but Mx > 0.3 was associated with poor clinical outcomes in traumatic brain injury patients [3]. Ten adults (six male, four female) with refractory SE and a mean age of 52 ± 16 years were included (Table 1). Initially, Mx was 0.30 ± 0.21 and did not significantly change during the measurement period (Fig. 1 Table 2). Four patients were extubated and transferred to the regular ward after less than four measurements. The collected data indicated that CA in patients with refractory SE after induction of analgo-sedation was intact and did not change in a relevant matter during the observational time. Moreover, Mx levels > 0.3 were not associated with poor clinical outcomes in the investigated cohort. Studies focusing on CA or CBFV in epilepsy patients are rare. In contrast to the presented data, a compromised CA was described in epilepsy patients in interictal states [1]. Furthermore, CBFV was increased during tonic-clonic seizures but was not observable during SE and nonconvulsive SE in comatose patients [4, 5]. Neither an increased CBFV nor alterations of CA were found; this may be attributable to sufficient seizure treatment due to analgo-sedation. In conclusion, cerebral hemostasis seems to be preserved in sufficiently treated patients with refractory SE.

**Table 1:** Physiological data

Age (years)	52 ± 16
Sex	6 male, 4 female
BMI (kg/m^2^)	27 ± 6
APACHE II	22.8 ± 3
GOS 4 + 5 (*n*)	6
GOS 2 + 3 (*n*)	3
GOS 1 (*n*)	1
ICU stay (days)	11 (9–18)

Parameters are expressed as mean ± standard deviation or median (minimum–maximum)

*APACHE II* Acute Physiology and Chronic Health Evaluation II, *BMI* body mass index, *GOS* Glasgow Outcome Scale, *ICU* intensive care unit

**Fig. 1:**
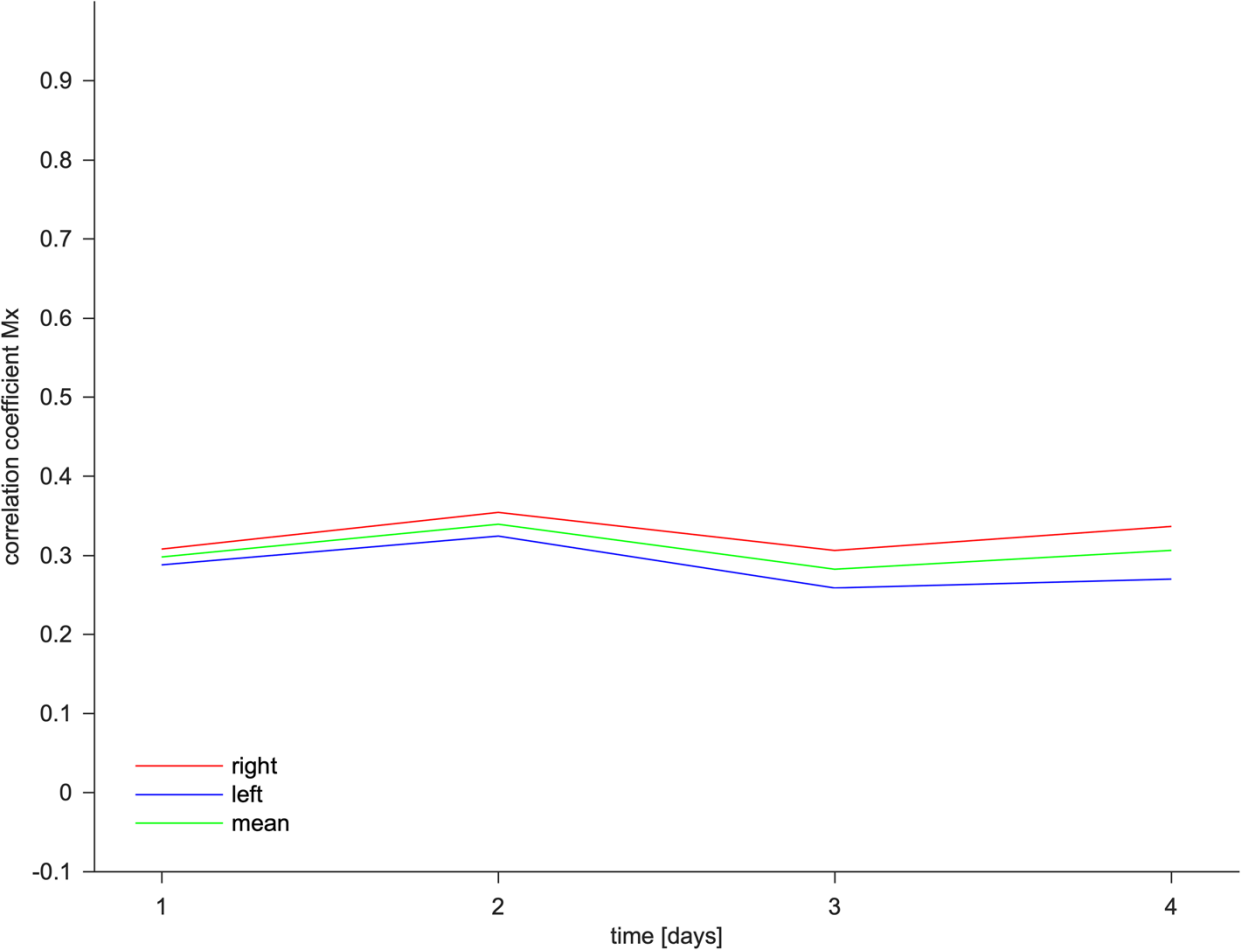
Time course of cerebrovascular autoregulation index. The figure presents the index of the cerebrovascular autoregulation (mean velocity index; Mx) during the first 4 days after onset of status epilepticus. The red line represents Mx of the right hemisphere, the blue line represents the left hemisphere, and the yellow line represents mean Mx

**Table 2:** Mean velocity index and clinical data

Day	*n*	Mx	SOFA	MAP (mmHg)	HR (beats/min)	CBFV_mean_ (cm/s)	PaO_2_ (kPa)	PaCO_2_ (kPa)
1	10	0.30 ± 0.21	10.5 ± 2.4	89 ± 14	65 ± 14	42 ± 15	20.5 ± 7.2	4.9 ± 0.7
2	9	0.34 ± 0.35	10.4 ± 1.6	84 ± 16	67 ± 23	55 ± 22	15.6 ± 2.4	5.1 ± 1.1
3	8	0.28 ± 0.34	10.0 ± 2.4	90 ± 11	74 ± 25	53 ± 23	14.4 ± 3.2	5.3 ± 0.9
4	6	0.32 ± 0.45	8.6 ± 4.6	97 ± 16	83 ± 21	56 ± 23	14.9 ± 2,8	6.9 ± 1.0

The number of patients with refractory status epilepticus is presented as count (*n*)

*CBVF*_*mean*_ mean cerebral blood flow velocity, *HR* heart rate, *MAP* mean arterial blood pressure, *Mx* mean velocity index, *PaCO*_*2*_ partial pressure of carbon dioxide, *PaO*_*2*_ partial pressure of oxygen, *SOFA* sequential organ failure assessment score

## Abbreviations

CA: Cerebrovascular autoregulation
CBFV: Cerebral blood flow velocity
Mx: Mean velocity index
SE: Status epilepticus
